# Alternative and aberrant splicing of human endogenous retroviruses in cancer. What about head and neck? —A mini review

**DOI:** 10.3389/fonc.2022.1019085

**Published:** 2022-10-20

**Authors:** Lorenzo Agoni

**Affiliations:** Unit of Gynecology and Obstetrics, Fondazione Poliambulanza Hospital, Brescia, Italy

**Keywords:** HERV human endogenous retroviruses, endogenous retroviruses (ERV), aberrant splicing, non-canonical splicing, non-conventional splicing, head and neck cancer, cancer

## Abstract

Human endogenous retroviruses (HERVs) are transcribed in many cancer types, including head and neck cancer. Because of accumulating mutations at proviral loci over evolutionary time, HERVs are functionally defective and cannot complete their viral life cycle. Despite that, HERV transcripts, including full-length viral RNAs and viral RNAs spliced as expected at the conventional viral splice sites, can be detected in particular conditions, such as cancer. Interestingly, non-viral–related transcription, including aberrant, non-conventionally spliced RNAs, has been reported as well. The role of HERV transcription in cancer and its contribution to oncogenesis or progression are still debated. Nonetheless, HERVs may constitute a suitable cancer biomarker or a target for therapy. Thus, ongoing research aims both to clarify the basic mechanisms underlying HERV transcription in cancer and to exploit its potential toward clinical application. In this mini-review, we summarize the current knowledge, the most recent findings, and the future perspectives of research on HERV transcription and splicing, with particular focus on head and neck cancer.

## Introduction

Human endogenous retroviruses (HERVs) are remnants of ancient retroviral infections integrated into the human genome. Because of recombination events over evolutionary time, most HERV loci are reduced to solitary Long Terminal Repeats (LTRs) (soloLTRs) or viral genomic fragments of various lengths. There are many different families of HERVs, and altogether, they comprise 8% of the human genome. Among these, the HERV-H family is the largest group, consisting of full-length elements (about 100 copies), fragmented elements (800–900 copies), and soloLTRs (about 1,000 copies) (1, 2). None of the full-length proviruses can complete their viral life cycle, due to mutations that accumulated at the provirus loci over evolutionary time, not even HERV-K, although it is the most recent retrovirus that infected the human germline and thus the most intact among all the HERVs. There are approximately 30 full-length HERV-Ks in the human genome out of the several hundreds of HERV-K loci detected, most of which contain fragments or soloLTRs (3). Although none of such full-length HERVs is any longer fully active as virus, transcription has been detected in particular conditions, such as cancer. RNAs from members of multiple HERV families, including HERV-H, HERV-K, HERV-W, HERV-E, HERV-P, HERV-T, HERV-F, HERV-R, and HERV-S, have been identified in cancer (4–13). HERVs have been found active as well as in other conditions such as autoimmune diseases (14, 15), particularly in Systemic Lupus Erythematosus (16, 17), in neurodegenerative diseases (18, 19), particularly in multiple sclerosis (18, 20, 21), and have implications with immunity and other viral infections (22), particularly with HIV (23, 24). Transcription at HERV loci may start from the viral promoter within the 5′LTR and from an upstream promoter. In fact, transcription has been detected also at heavily rearranged and mutated HERV genomic fragments, even in the absence of a functional viral promoter (25–27). The role of such transcripts, often part of long non-coding RNAs (lncRNAs), and their function as RNAs or potential for translating into proteins are largely unknown. However, some viral fragments have been highly conserved through evolution and the derived translated protein exploited by the host for its own purposes. The most notable example is Syncytin-1 that is a protein encoded by ERVW-1 gene, a remnant of an ancient HERV-W *env* gene and that is a fundamental protein for placenta formation in humans. On the other hand, translation of canonical viral proteins (Gag, Pro, Pol, and Env) from full-length HERV-K loci has been described. It is relevant to point out that some HERV-K proteins (Env and accessory proteins Rec and Np9) are translated from spliced viral transcript. In an effort to better characterize full-length HERV-K transcription at specific proviral loci, in our previous work (28), we detected transcripts derived from aberrant alternative splicing events, across different cancer types, including head and neck cancer cell lines.

## Human endogenous retroviruses are, in essence, retroviruses

Endogenous retroviruses (ERVs), including HERVs, follow the same replicative life cycle of all retroviruses, with the specific characteristic of infecting the genome of germ cells and thus integrating into the genome of the host and being passed to the progeny.

The provirus, which is the integrated DNA form of the retrovirus, is composed of an internal genome segment containing the viral protein coding genes and an LTR at each end of the viral genome. Enhancers and promoters and initiation start site for viral transcription are located in the LTR.

The retroviral genome consists of at least four basic genes: *gag*, *pro*, *pol*, and *env*. These genes encode structural and enzymatic proteins that are essential for viral replication, including the viral capsid components (*gag*); protease (*pro*); reverse transcriptase (RT), ribonuclease H, and integrase (*pol*); and the envelope protein (*env*). Many, but not all, retroviruses have additional genes that encode accessory proteins, such as *rec* in HERV-Ks. For almost all retroviruses, some of these genes (*gag*, *pro*, and *pol*) are translated from the primary mRNA transcript. For the other viral genes including *env*, one or more splicing events must occur.

## Death of the virus: HERVs are now viral remnants

ERVs, once stably integrated in the host genome in the form of DNA proviruses, have to face several challenges to survive. Mutations and other recombination events inevitably accumulate at these loci over evolutionary time, just as they do at any host locus. Because, *per se*, there is no selection pressure on the host to maintain the ERV proviruses in an intact form, with certain exceptions at least for parts of certain proviral genomes, these will eventually include lethal mutations that prevent each ERV from encoding new, fully infectious viral particles and thus the genetic death of that element.

These mutagenic events include nucleotide substitutions, deletions, insertions, and translocations. One particularly important and common type of event for ERVs is the recombination between two homologous LTRs in the same provirus, leading to a soloLTR, with one LTR and the internal viral genome being excised. Even point mutations can irremediably inactivate a provirus if they compromise the function of essential viral proteins.

Thus, to date, no individual HERV-K provirus present in the human genome has been found to retain a full capability of encoding an infectious genome capable of completing the viral life cycle.

## Life in death: HERVs are transcribed, nonetheless

HERV expression is regulated at the level of the LTRs, which function as promoter and contain the transcription starting site and numerous transcription factor binding sites (29, 30). Because HERVs infect the germline, it is reasonable that specific transcription factors that are active during oogenesis, spermiogenesis, or early embryo development are exploited for viral activation. Such factors have not been clearly characterized yet (31, 32). In addition to the viral life cycle, HERVs have been detected in several physiologic and pathologic conditions (33–37). Thus, the LTR is likely to be able to bind also with transcription factors other than those specifically active in germline (30).

The implications of transcription of heavily mutated or rearranged HERV genes are mostly unknown. HERVs may translate for viral proteins, which are mostly non-functional as explained above, truncated viral proteins, and non-viral proteins resulting from frameshift mutations, indels, or various genetic rearrangements. In addition, transcripts from these loci may lack obvious Open Reading Frame (ORF).

However, most HERV loci are soloLTRs. In such cases, the virus no longer exists at the specific genomic locus, and the soloLTR constitutes a relic of the previous infection (38). Nonetheless, sequences within the LTR may still be usable by the host transcription factors. In fact, LTRs can serve as promoters in both sense and antisense orientations (39). Thus, they can influence the expression of nearby host genes (27, 40, 41). Moreover, soloLTR may promote transcription of long intergenic non-coding RNAs (lincRNAs) (42). The *SAMMSON* lncRNA is promoted by a soloLTR (LTR1A2) and was recently reported as involved in oncogenesis in melanoma (43). Other examples of LTR-promoted oncogenic lncRNAs include HULC in hepatocarcinoma (44), UCA1 in urotelial carcinoma (45), and LCT13, which is another lncRNA that is promoted by an antisense soloLTR (L1PA2), in colorectal cancer (46).

To avoid potentially deleterious transcription, both from soloLTRs and full-length proviruses of fragments, it is commonly assumed that HERVs are mostly silenced by epigenetic regulation (47, 48). Multiple strategies are used to regulate HERV transcription such as localization of soloLTRs and proviruses to heterochromatin and CpG methylation and histone deacetylation (49).

## HERVs in cancer and other conditions: Friend or foe?

Although HERVs’ role in health and disease has been explored for many years, it remains poorly understood (33–37).

An oncogenic role for HERV-K has been proposed. In fact, HERV-K transcripts and proteins have been detected in several cancer types, including teratocarcinoma (50–55), trophoblastic tumors and germ cell tumors (56, 57), seminomas (58–60), breast cancer (61–67), prostate cancer (68–71), leukemias (72–77), renal cancer (78), ovarian cancer (79–81), cervical cancer (28, 82), melanoma (83–87), soft tissue sarcoma (88), osteosarcoma (89), Kaposi’s sarcoma (90), glioblastoma (91), astrocytic tumors (92), hepatoblastoma (93, 94), and hepatocellular carcinoma (95).

The correlation between HERV-K expression and cancer is not sufficient in itself to clarify whether HERV-K has tumorigenic activity. In fact, overexpression of Rec or Np9 in transgenic mice has been shown to cause tumors (96).

HERV-K expression has also been detected in normal tissues and physiological conditions. In the human placenta, in addition to the abovementioned HERV-W and HERV-FRD, HERV-K has been detected as well (73, 97), but its role in physiology or disease, if any, has not been elucidated yet. In fact, HERV-K has been speculated to have a role in placental dysfunctions such as preclampsia (98). HERV-K viral particles can be detected by electron microscopy in normal human placenta (36).

As abovementioned, the well-conserved *env* genes from HERV-W1 and HERV- FRD transcribe for Syncytin-1 and Synicitin-2, respectively, and mediate the fusion of villous cytotrophoblasts during placentation (99, 100). In fact, Env protein has fusogenic properties and has been proposed to have a role in cancer, including in epithelial-to-mesenchymal transition (101, 102).

Moreover, *env* genes have been proposed to exhibit an immunosuppressive role that is important for preventing maternal rejection of the semi-allogenic fetus during pregnancy (103). Such property has been speculated to be exploited by cancer cells as well to facilitate tolerance.

HERV-H has been detected in human normal tissue, specifically in placenta, and cancer, including breast cancer (104), pancreatic cancer (105), liver cancer (106, 107), prostate cancer (108), ovarian cancer (109), lung cancer (110), colon cancer (111, 112), cervical cancer (104), and others (2, 107, 113).

An interesting regulatory role for pluripotency in human embryonic stem cells and induced pluripotent stem cells has been described for HERV-H, including ncRNAs, enhancers, and alternative promoters, and markers of topologically associating domain (TAD) boundaries (114).

Little is known regarding HERV-K and HERV-H transcription in normal tissues other than placenta.

Bioinformatic search for HERV-conserved ORFs led to the identification of a small but interesting number of *env*-related genes with a full-length coding sequence, such as syncytin-1 and syncytin-2 (115, 116).

Recent research led to the identification of a peculiar HERV locus transcribing for a unique Env protein, called HEMO, which is released in the human blood circulation. HEMO has also been detected in placenta and various cancers as well, including ovarian cancer and endometrial cancer, and in germline, liver, lung, or breast tumors (117). The HEMO retroviral env gene belongs to the MER34 family, which comprise only highly degenerated and rearranged elements.

Little or none is known about the vast world of HERV-containing lncRNAs. Several examples have been described but functions and significance is mostly unknown (118–120).

One example is lncMER52A, a liver cancer–specific oncogenic lncRNA transcribed by MER52A LTR retrotransposon of the ERV1 class (121).

Another example is the overexpression in breast cancer of the TROJAN lncRNA, which contains a complete LTR70 sequence of several mosaic LTRs flanked by MER67C and LTR56 (122).

## The good, the bad, and the ugly: Canonical, alternative, and aberrant splicing of HERV transcripts

Intact full-length HERVs produce a single full-length RNA transcript, from 5′LTR to 3′LTR. Then, a single splicing event must occur to produce the 1X-spliced *env* transcript. An additional splicing event is needed to produce the 2X-spliced *np9* and *rec* transcripts in HERV-Ks.

In the previous paragraph, we list the many examples of detection of Env, Rec, and Np9 proteins in cancer. All of them must have derived from canonical splicing events of the HERV full-length transcript. Splicing occurs when specific splicing signal sequences, namely, splice donor (SD) and splice acceptor (SA), are recognized by the spliceosome machinery, and intronic sequence is excised. Typically, only a fraction of HERV full-length transcript undergoes splicing, because the full-length transcript constitutes the genome for the forming virions.

It has been speculated that the highly mutated HERV genome may disrupt canonical SD/SA signals and allow cryptic splicing sites to emerge. In fact, many of such sites have been identified throughout the HERV genome (123, 124).

**Figure 1:**
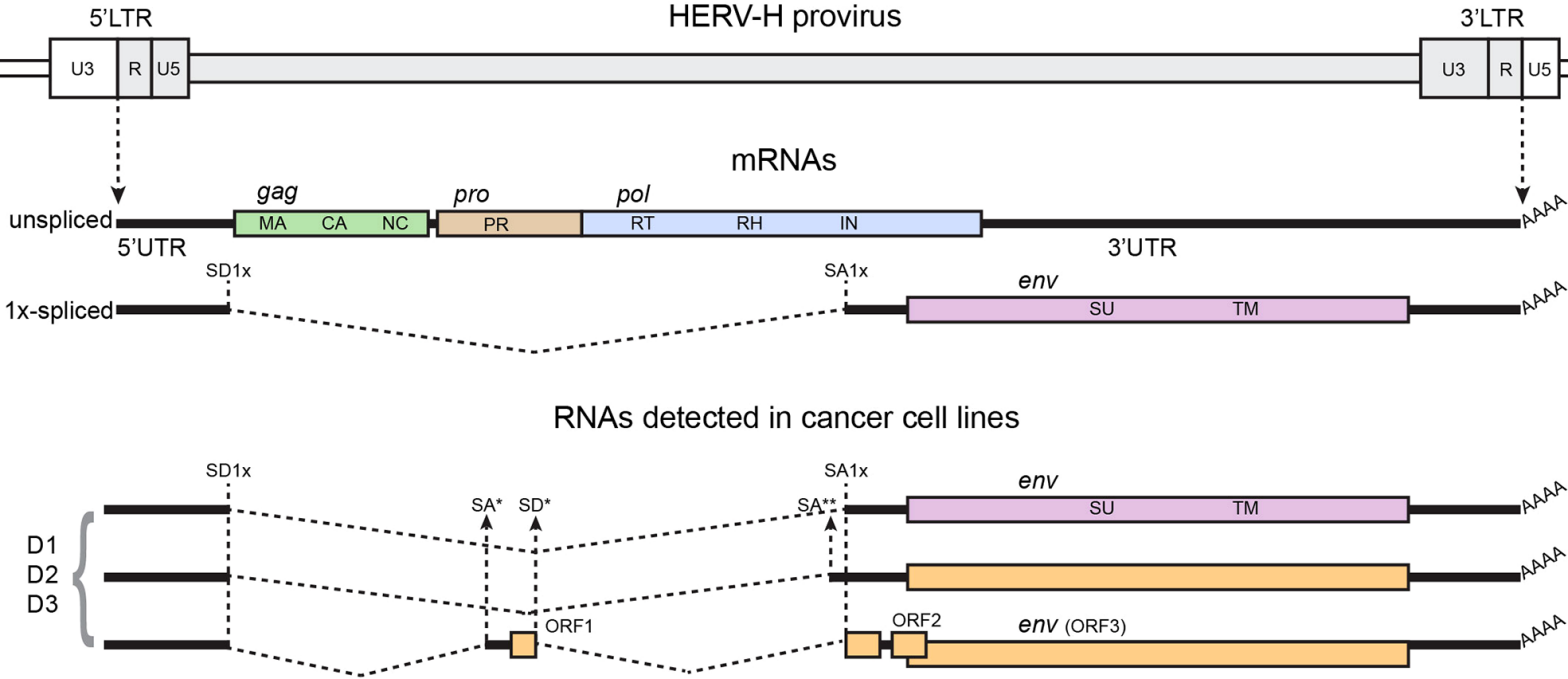
Structure of the HERV-H genome, canonical spliced mRNAs, and spliced RNAs detected in (113), from which the figure was adapted. A genetic map of a HERV-H provirus (gray) inserted into flanking host genome sequences is shown. The unspliced primary viral transcript and singly spliced env mRNA are shown below the viral genome. Poly (A) tails are indicated (AAAA). The dashed, angled line shows the excised intronic sequences. Colored boxes indicated the different genes, indicated in the figure by name and ORF. Splice donor (SD) and splice acceptor (SA) sites are indicated on map, including canonical sites for 1x-spliced mRNA (SD1x and SA1x) and canonical sites for 2x-spliced mRNAs (SD2x’ for type I proviruses, SD2x” for type II proviruses, and SA2x), and individual alternative SD/SA are identified by stars (*, **). Black vertical dashed lines identify canonical SD/SA sites across the panel; black arrows show alternative SD/SA sites. D1, D2, and D3 are fictitious names of individual HERV-H viruses detected in the study.

Previous work from Lindeskong and Blomberg (113) has shown alternative splicing for HERV-H *env* transcripts from normal and leukemia lymphocytes (**Figure 1**). These alternative splicing sites use canonical consensus signals for major spliceosome. Although the function and significance of such findings is unknown, it can be speculated that these constitute cryptic SD/SA sequences. The shift to these cryptic signals was not due to mutations at the canonical splice sites, as the functional canonical splice sites could be sequenced. In fact, multiple SD and SA signals have been predicted in HERV-H by bioinformatic analysis (125). Moreover, normal and leukemia lymphocytes showed different levels of amplification of the spliced *env* transcripts, thus indicating that the cellular type could determine which alternative splicing event to favor. Thus, the molecular mechanisms for the SD/SA choice were not identified. It is relevant to point out that some of these transcripts have ORFs that could possibly be translated into novel proteins, not *per se* related to the original viral proteins.

**Figure 2:**
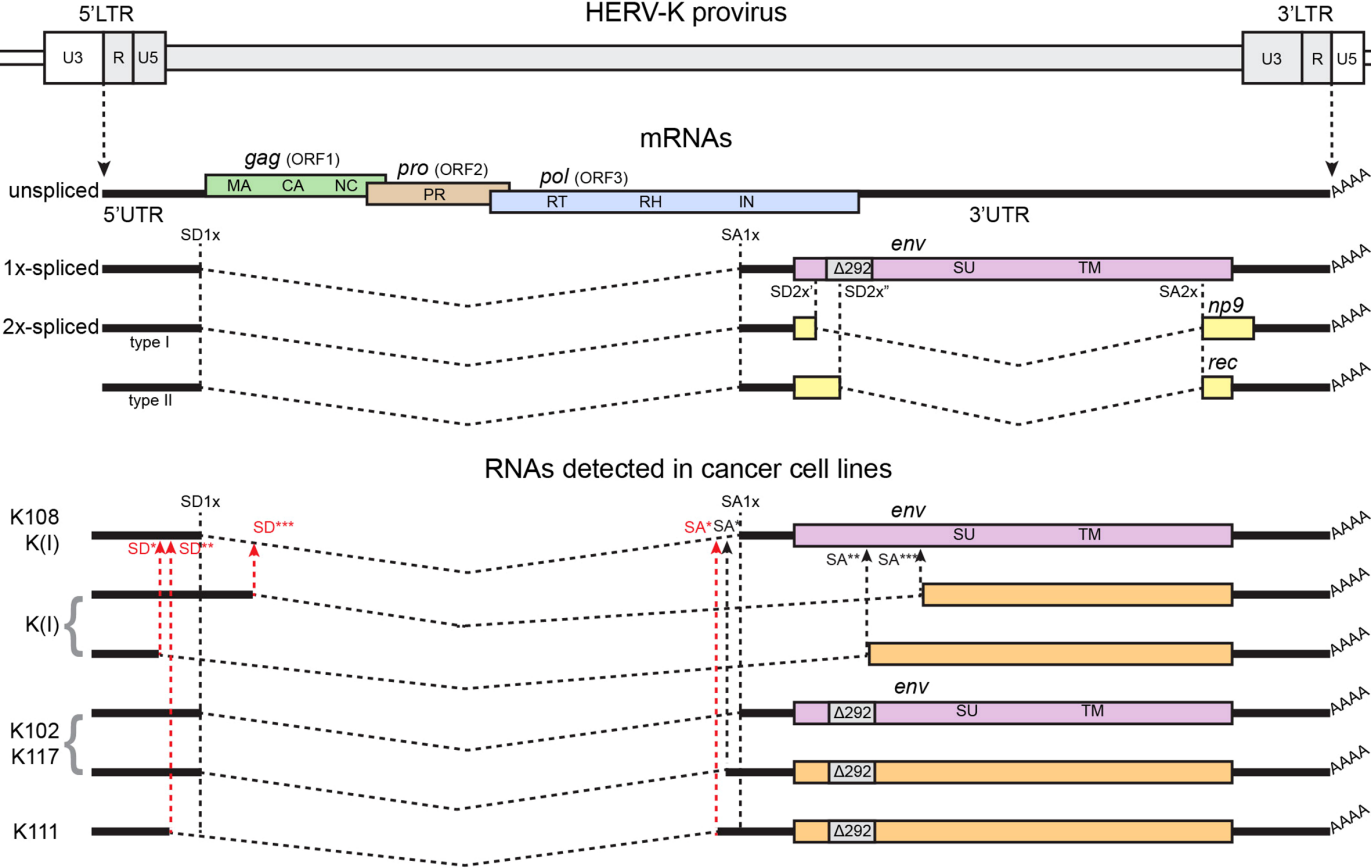
Structure of the HERV-K genome, canonical spliced mRNAs, and spliced RNAs detected in (28), from which the figure was adapted. A genetic map of a HERV-K provirus (gray) inserted into flanking host genome sequences is shown. The unspliced primary viral transcript, singly spliced env mRNA, and doubly spliced rec and np9 mRNAs are shown below the viral genome. Poly (A) tails are indicated (AAAA). The 292 nucleotide deletion of type 1 HERV-K proviruses spanning the pol-env junction is indicated (Δ292). The dashed, angled line shows the excised intronic sequences. Colored boxes indicated the different genes, indicated in the figure by name and ORF. Splice donor (SD) and splice acceptor (SA) sites are indicated on map, including canonical sites for 1x-spliced mRNA (SD1x and SA1x) and canonical sites for 2x-spliced mRNAs (SD2x’ for type I proviruses, SD2x” for type II proviruses, and SA2x), and individual alternative SD/SA are identified by stars (*, **, and ***) and are written in red in case of aberrant sites, without canonical sequences for spliceosomes. Black vertical dashed lines identify canonical SD/SA sites across the panel, black arrows show alternative SD/SA sites, and red arrows show SD/SA aberrant sites. HERV-K108, HERV-K(I), HERV-K102, HERV-K117, and HERV-K111 are the names of individual HERV-Ks detected in the study.

With the purpose of screening for viral splicing transcripts in cancer cell lines, in our previous work (28), we undertook RT-PCR across splicing sites of full-length HERV-K in various cancer cell lines, including breast cancer, cervical cancer, prostate cancer, and head and neck cancer, which are expected to find 1X-spliced transcript for *env* and 2X-spliced transcript for *np9* or *rec*, respectively, for type I and type II HERV-K-HML2 viruses (25). Indeed, the spliced transcripts were detected in almost all cancer cell lines across the tested panel (**Figure 2**). Sequencing of the RT-PCR products was performed to identify the specific loci of origin of the transcripts. These analyses identified a total of seven different individual HERV-K loci among the 12 cell lines tested: HERV-K102, HERV-K108, K(I), HERV-K106, HERV-K107, HERV-K111, and HERV-K117.

Unexpectedly, while most RT-PCR products showed splicing at the expected positions within the HERV-K genome, the transcripts spliced at the additional previously unidentified sites were also detected (**Figure 2**). The conventional 1X-env splicing sites were detected for HERV-K108, HERV-K109, HERV-K(I), HERV-K102, and HERV-K117. Four loci—HERV-K102, HERV-K(I), HERV-K117, and HERV-K111—showed unusual 1X-env splicing variants formed from the use of alternative SD/SA sites. The splice sites that were detected in some instances matched the consensus signals for the major or minor spliceosomes, whereas, in other instances, they did not. For HERV-K102, the same splice sites were detected in six different cell lines, and for HERV-K117, the same sites were detected in two. Such aberrant spliced transcripts were detected in the majority of the cell lines, and in some instances, both the conventional and aberrant splice sites were detected for particular proviruses [HERV-K102, HERV-K(I), and HERV-K117].

This finding was unexpected, and the possible biological significance and the underlying molecular mechanism are unknown.

## What about HERVs in head and neck cancer?

Although HERV expression has been detected in many cancer types, in head and neck cancers, it has been rarely tested.

The most relevant example comes from Kolbe et al. (126) who analyzed 43 paired tumor and adjacent normal tissue samples from The Cancer Genome Atlas program. Transcripts were detected from over 3,000 specific HERV loci, in tumor and adjacent normal tissue. Approximately one-third of them were differentially expressed between the two tissue types. Most differentially expressed HERVs showed higher levels in tumor tissue. Differentially expressed HERVs were enriched in members of the HERV-H family. A hierarchical clustering based on HERV expression was performed, and the two resulting distinct clusters showed significant difference in survival.

Although this study was performed by looking at the single loci level, a clustering algorithm was used to generate two prognostic distinct clusters. Thus, the contribution of specific HERV loci to survival was not investigated—not the details at the molecular level regarding which portion of the viral genome and genes were transcribed and whether any splicing event occurred.

Cuffel et al. (127) studied the expression of cancer-testis and other tumor-associated antigens in head and neck squamous cell carcinoma (HNSCC). Samples from 57 HNSCC patients were analyzed by RT/PCR, Immunohistochemistry (IHC), and correlated with survival. Among the results, it is relevant to highlight that a HERV-K–related antigen, HERV-K-MEL, was among the most frequently expressed genes as it was detected in 42% of the patients. However, in their analysis, HERV-K-MEL expression did not impact survival. HERV-K-MEL is an HERV-K env-related antigen that was first identified as recognized by cytolytic T lymphocytes in melanoma (84).

A recent study by Zapatka et al. (128) has shown ERV1 expression in HNSCC. The authors, as part of the Pan-Cancer Analysis of Whole Genomes Consortium, performed a whole-transcriptome sequencing data from over 2,000 cancer samples across 38 tumor types. Among these, HERV expression, particularly ERV1, was detected in HNSCC, although no correlation with survival was evident.

Michna et al. (129) reported the expression of ERVMER34-1 and ERV3-1 (HERV-R) in CAL-33 HNSCC cell line. Interestingly, ERV3-1 was upregulated in response to irradiation. The authors speculated that these findings may have implications in radiosensitivity in cancer.

Landriscina et al. (130) have shown that RT is active in FRO, WRO, and ARO human thyroid carcinoma cell lines. They showed that nevirapine and efavirenz, two RT inhibitors that are usually employed in HIV treatment, reversibly inhibit cell proliferation in the undifferentiated thyroid carcinoma ARO and FRO cells. However, they did not characterize RT origin. In fact, in addition to HERVs, most retroelements, including LINE elements, have an RT coding gene (131).

Indeed, HERV-K is expressed in head and neck cancer cell lines, and splicing events are detected.

In our abovementioned study (28), we tested the head and neck cancer cell lines FaDu, UPCI-SCC-90, and UM-SCC-47. 1X-env and 2X-rec transcripts were detected in all three cell lines, whereas 2X-np9 was only detected in UPCI-SCC-90. Specifically, HERV-K102, HERV-K108, K(I), and HERV-K106 were detected. In all three cell lines, aberrant 1X-env transcripts were detected: HERV-K(I) in UPCI-SCC-90, and HERV-K102 in FaDu and UM-SCC-47.

To the author’s knowledge, no other examples of HERV detection in head and neck cancer appear available in the scientific literature.

## Discussion

HERV activation is a common feature in cancer. However, its role, if any, has not been fully elucidated yet.

Although HERVs are highly mutated, they have been shown to retain, at least in some cases, enough viral functions to translate to the canonical viral proteins, including those that require splicing events. Moreover, alternative and aberrant splicing variants have been detected.

Pre-mRNA splicing is a common post-transcriptional event for both the host cell and retroviruses, including HERVs. There are several different types of alternative splicing, among which the most common include exon skipping, selection of splice donor, and selection of splice acceptor. Alternative splicing allows to expand the variety of encoded proteins, but it may also be involved in regulation of translation, for example, by including an early stop codon. The splicing process involves the spliceosome machinery, which is formed by five small nuclear ribonuleoproteins and several proteins. The cell has two different types of spliceosome: the U2-type “major” spliceosome and the U12-type “minor” spliceosome. They recognize different specific intronic sequences for the splice sites. Most splicing occurs through the major spliceosome. Alternative and aberrant splicings have been involved in cancer initiation and progression (132).

Transcription of the HERV provirus, by RNA polymerase II, generates a primary viral RNA that is both 5′ capped and 3′ polyadenylated, as any other mRNA of the host cell (124). Similarly to the host RNA, the primary RNA can be regulated by internally m^6^A and m^5^C methylations (133).

The primary transcript translates the *gag-pro-pol* genes into capside (MA, CA, and NC), protease (Pro), RT, and integrase (IN) proteins and constitutes the genome for the new virions. Env requires a splicing event to occur, and the accessory proteins Rec and Np9 require a second splicing event. Both alternative splicings require simple intron excision. Thus, the primary RNA must escape splicing to reach packaging into the forming virions. How this selection between splicing and packaging is made is unclear. It has been speculated that this may depend on splice site efficiency. In fact, the spliceosome machinery would intercept every single primary RNA transcript displaying splice sites with high efficiency, thus entirely preventing full-length RNA packaging. Conversely, the presence of low-efficiency splice sites would allow both RNA forms to co-exist. This ensures the correct life cycle of the retrovirus, but it may also facilitate alternative, cryptic, splice sites to emerge and be utilized. This may explain the findings of alternative splicing of HERV-H (113) and HERV-K (28) of the splice site selection type. Similar mechanisms have been observed for HIV (134) and other retroviruses (124). However, some splice site sequences in HERV-K alternative splicing do not match with signal sequences for either U2- or U12-type spliceosomes. This observation is unexpected and unexplained.

The alternative and aberrant splice sites detected highlight the possibility of new research in the field of spliceosome functions. In fact, the sequences of most of the alternative sites that were detected shared only partial overlap with those of the conventional splicing signals. What determinants within the viral genome affect the utilization of alternative splice sites is currently unknown, and further studies may shed light on the basic mechanisms of splicing.

The use of non-canonical SA/SD sequences has been shown to some extent in trans-splicing (135), which is an unusual form of splicing between two individual pre-RNA transcripts. Most trans-splicing is expected to follow the canonical spliceosome-mediated splicing process. However, a transfer RNA (tRNA)-mediated splicing, which does not require the canonical consensus sequences for splice sites nor the spliceosome machinery, has been described, at least for trans-splicing (136).

The potential for translating into protein may vary. In fact, the assumption that a viral transcript will translate into a viral protein must be verified, especially for HERVs that carry numerous mutations in their sequence and that not only may affect viral protein function but also may prevent correct polypeptide three-dimensional conformation. In fact, HERV transcripts may be part of lncRNAs, by polymerase read-through transcription of proviral loci (137). Such transcripts may bear regulatory functions, particularly on gene expression (138). lncRNAs may interact with DNA, RNA, or proteins: lncRNAs may promote or repress transcription, by working as signals or decoys, respectively, or may function as epigenetic regulators or even as scaffolds by interacting with various protein partners (139–141). The inclusion of HERV components into lncRNAs has been reported (142).

HERVs’ proteins and transcripts constitute an attractive target for therapy. Many authors have been exploiting such knowledge to refine various strategies against HERVs to selectively target cancer cells (84, 103, 143–145).

The need for locus-specific analyses is evident. However, the high degree of repetitive and highly similar sequences in HERV elements has made locus-specific characterization of HERVs a significant challenge.

Although HERVs have been detected in head and neck cancer, the research in this cancer type is still at a very early stage and warrants further study.

## Author contributions

LA has conceived, performed literature research for, and written the article. The author confirms being the sole contributor of this work and has approved it for publication.

## Conflict of interest

The author declares that the research was conducted in the absence of any commercial or financial relationships that could be construed as a potential conflict of interest.

## Publisher’s note

All claims expressed in this article are solely those of the authors and do not necessarily represent those of their affiliated organizations, or those of the publisher, the editors and the reviewers. Any product that may be evaluated in this article, or claim that may be made by its manufacturer, is not guaranteed or endorsed by the publisher.
